# Pollution Characterization and Environmental Impact Evaluation of Atmospheric Intermediate Volatile Organic Compounds: A Review

**DOI:** 10.3390/toxics13040318

**Published:** 2025-04-19

**Authors:** Yongxin Yan, Yan Nie, Xiaoshuai Gao, Xiaoyu Yan, Yuanyuan Ji, Junling Li, Hong Li

**Affiliations:** 1 State Key Laboratory of Environmental Criteria and Risk Assessment, Chinese Research Academy of Environmental Sciences, Beijing 100012, China; yyx_in@163.com (Y.Y.); nieyan80@outlook.com (Y.N.); s202265286@emails.bjut.edu.cn (X.G.); yanxiaoyu22@mails.jlu.edu.cn (X.Y.); ji.yuanyuan@craes.org.cn (Y.J.); 2 Department of Environmental Science and Engineering, Beijing University of Technology, Beijing 100124, China; 3 College of Earth Science, Jilin University, Changchun 130061, China

**Keywords:** intermediate volatile organic compounds (IVOCs), pollution characterization, contributions to SOAs, human health risk assessment, systematic review

## Abstract

Atmospheric intermediate volatile organic compounds (IVOCs) are important precursors of secondary organic aerosols (SOAs), and in-depth research on them is crucial for atmospheric pollution control. This review systematically synthesizes global advancements in understanding IVOC sources, emissions characterization, compositional characteristics, ambient concentrations, SOA contributions, and health risk assessments. IVOCs include long-chain alkanes (C_12_~C_22_), sesquiterpenes, polycyclic aromatic hydrocarbons, monocyclic aromatic hydrocarbons, phenolic compounds, ketones, esters, organic acids, and heterocyclic compounds, which originate from primary emissions and secondary formation. Primary emissions include direct emissions from anthropogenic and biogenic sources, while secondary formation mainly results from radical reactions or particulate surface reactions. Recently, the total IVOC emissions have decreased in some countries, while emissions from certain sources, such as volatile chemical products, have increased. Ambient IVOC concentrations are generally higher in urban rather than in rural areas, higher indoors than outdoors, and on land rather than over oceans. IVOCs primarily generate SOAs via oxidation reactions with hydroxyl radicals, nitrate radicals, the ozone, and chlorine atoms, which contribute more to SOAs than traditional VOCs, with higher SOA yields. SOA tracers for IVOC species like naphthalene and β-caryophyllene have been identified. Integrating IVOC emissions into regional air quality models could significantly improve SOA simulation accuracy. The carcinogenic risk posed by naphthalene should be prioritized, while benzo[a]pyrene requires a combined risk assessment and hierarchical management. Future research should focus on developing high-resolution online detection technologies for IVOCs, clarifying the multiphase reaction mechanisms involved and SOA tracers, and conducting comprehensive human health risk assessments.

## 1. Introduction

Atmospheric pollution remains one of the major environmental challenges facing the world [1]. Currently, atmospheric fine particulate matter (PM_2.5_) pollution is an important factor hindering air quality improvement in many countries, significantly impacting human health, the atmospheric environment, and the global climate [2]. Organic aerosol (OA) is an important composition of PM_2.5_, accounting for 20–90% of PM_2.5_ [3]. Secondary organic aerosol (SOA) is particulate organic matter formed from gaseous organic compounds through atmospheric chemical reactions. It has been reported that atmospheric SOAs account for 64–95% of OAs in North American cities, while they account for 44–71% of OAs produced during urban haze events in China, and the contribution rate of SOAs to OAs shows a year-by-year increasing trend in some areas [1,4]. However, traditional models often underestimate SOA levels compared to field observations because the types of precursors that contribute significantly to SOAs have not been fully identified at present [5,6]. Therefore, there are still many challenges in regard to accurately modeling and predicting the generation process for SOAs and implementing precise control actions over its precursors.

In recent years, both field observations and laboratory studies have confirmed that the gas-phase precursors of SOAs include volatile organic compounds (VOCs), intermediate volatile organic compounds (IVOCs), and semi-volatile organic compounds (SVOCs). Based on the semi-empirical saturation concentration (C*) of the volatility basis set (VBS), it is considered that compounds with a C* > 10^6^ μg m^−3^ are VOCs, compounds with 10^3^ μg m^−3^ < C* < 10^6^ μg m^−33^ are IVOCs, and compounds with 1 μg m^−3^ < C* < 10^2^ μg m^−3^ are SVOCs. The saturated vapor concentrations, i.e., volatility, of IVOCs are in between those of VOCs and SVOCs, with volatility ranges comparable to C_12_–C_22_ n-alkanes [7,8]. IVOCs typically exist in a gaseous state at ordinary temperature, but can also be partially present in the particulate phase. They can participate in particulate-phase reactions through gas–particle partitioning to form species with lower volatility [7,9]. The composition of IVOCs is highly complex. Limited by the existing technological means, only some compositions, such as n-alkanes and polycyclic aromatic hydrocarbons (PAHs), have been identified, while a large number of compositions have not yet been identified at the molecular level, including numerous cycloalkanes and branched alkanes. There is a paucity of reports on these unresolved complex mixtures (UCMs) [10,11].

Research on IVOCs is becoming increasingly multidimensional and refined. In regard to field observations, the focus of the research is on accurately calculating the emissions of IVOCs and deeply analyzing their pollution sources, in order to comprehensively understand their actual performance in the atmospheric environment [12]. In laboratory simulations, the oxidation pathways, product compositions, yields, and yield modifiers of IVOCs in the formation of SOAs have been focused on, striving to reveal the complex reaction mechanisms of IVOCs [13,14]. In model simulations, the introduction of IVOC-related parameters has significantly enhanced the accuracy of atmospheric simulations, providing a strong theoretical basis for in-depth analysis of the pathways and contribution ratios of IVOCs during the SOA formation process [12]. In terms of environmental health, research has focused on assessing the exposure levels of IVOCs in the population and systematically evaluating potential health risks, with the goal of providing a scientific basis for public health protection [15]. Meanwhile, new detection methods are continuously emerging (such as Comprehensive Two-dimensional Gas Chromatography, GC×GC) to address the challenges in regard to IVOC species identification, and their precision is gradually improving [16].

Although the environmental concentrations of IVOCs are usually lower than those of routinely monitored VOCs, some IVOC compositions and their oxidation products can lead to chronic toxic effects through inhalation exposure and skin penetration. The toxicity mechanisms involve multiple pathways, including DNA damage, oxidative stress, and epigenetic regulation; therefore, the health risks should not be overlooked [15,17,18]. For instance, as a significant component in the composition of IVOCs, PAHs are recognized as one of the most important pollutants, due to their carcinogenicity, toxicity, and mutagenicity, posing a serious threat to human health [19]. Thus, conducting a health risk assessment of IVOC species can help identify high-risk IVOC species, fill the current gaps in the knowledge on IVOC pollutants, and enable more effective measures to reduce their negative impacts on public health and the ecological environment.

Recent research has revealed the important role of IVOCs on environmental and human health. However, the current understanding of IVOCs remains in its infancy, due to limitations in terms of the measurement technology and insufficient research on their chemical mechanisms. These limitations not only hinder accurate assessments of the environmental health risks posed by IVOCs, but also affect the ability of various parties to develop effective pollution control strategies. Therefore, a comprehensive understanding of the emission characteristics, oxidation mechanisms, and toxicity risks of IVOCs is crucial for precise source apportionment, emission control, improving atmospheric pollution governance, and uncovering potential health risks.

Research on IVOCs has made some progress. Tan et al. summarized major IVOC measurement techniques for use in the atmosphere [20]. Tang et al. focused on IVOC sources and their contributions to SOAs [21]. Wang et al. reviewed IVOC definitions, measurement techniques, field observation results, and laboratory and model simulation findings, and emphasized the significant contribution of IVOCs to SOAs [22]. Ling et al. provided insights into the key role of IVOCs in SOA formation and the impact mechanisms [23]. Kumar and Akinrinade examined IVOC health risks in southern California in the USA and India [18,19]. The existing review articles show clear differences in the research focus. For example, studies on the contribution of IVOCs to SOA formation vary, with some focusing on the reaction mechanisms and others on observational data analysis. These differences limit the systematic understanding of IVOCs. Additionally, most current research links IVOCs with SVOCs, and there are only a few independent studies on IVOCs. Furthermore, there is a lack of comprehensive summaries on the toxic effects and health risks of IVOCs.

Adopting an innovative approach based on the full-chain research results for “emission–exposure–transformation–risk”, this article collects a large amount of field observation data, smog chamber experimental results, and chemical transport model simulations on IVOCs, sorts out the latest domestic and international research progress, systematically summarizes IVOC source emission characteristics, ambient concentrations, SOA formation contributions, and so on, and conducts health risk assessments of typical IVOC species using the data reported in this paper. The findings of this review answer scientific questions about the characterization of IVOC emissions, the distribution of ambient concentrations, their contribution to SOA production, and their risk to human health. It can provide a scientific basis for the refined management and control of IVOCs and promote a paradigm shift in atmospheric pollution control, from “total amount control” to “reactive species priority” and “health risk priority”.

## 2. Methodology

### 2.1. Method of Systematic Review

**Figure 1:**
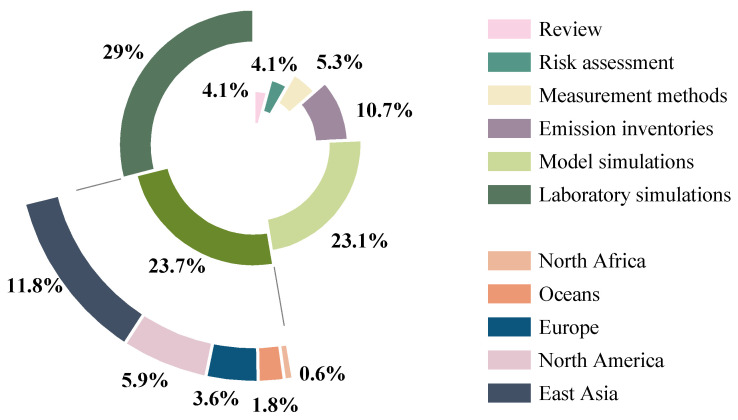
The proportion of different types of papers collected. The grass-colored green section in the figure represents field observation papers. The arcs represent the distribution of observations from different sites in the field observation studies.

In this study, 151 valid articles were obtained through a search using the core terms “intermediate volatile organic compounds (IVOCs)”, “polycyclic aromatic hydrocarbons (PAHs)” and “risk assessment” in the Web of Science and China Knowledge Network (CNKI) databases, and excluding non-peer-reviewed papers and conference abstracts. These articles cover reviews, emission inventories, field observations, lab simulations, model simulations, risk assessments, and detection methods, and the geographical scope of the field observations spans East Asia, North America, Europe, North Africa, and oceans, forming a full-chain evidence system for “emission–exposure–transformation–risk” (Figure 1).

### 2.2. Method of Human Health Risk Assessment

This study assessed the health risks of IVOCs based on observational data from published papers. The assessment used the inhalation exposure-based method recommended by the Risk Assessment Guidance for Superfund Volume I: Human Health Evaluation Manual (Part F, Supplemental Guidance for Inhalation Risk Assessment) (EPA-540-R-070–002) from the U.S. Environmental Protection Agency (EPA) and the Technical Specifications for Health Risk Assessment of Ambient Air Pollution (WS/T 666–2019) published by the Chinese National Health Commission [24,25]. It assessed the carcinogenic and non-carcinogenic risks of selected IVOC species, so as to comprehensively evaluate the potential impacts of IVOCs on human health via the inhalation route. The relevant parameters were from the Integrated Risk Information System (IRIS, https://www.epa.gov/iris, accessed on 6 April 2025) and the Exposure Factors Handbook of the Chinese Population: Adults.

Based on the IRIS, this article will first identify the IVOCs that pose health risks to humans from the existing research, prioritize them in regard to risk assessments, and compile toxicity data for these compounds. Second, it will present exposure data and estimate exposure levels. Finally, using ambient IVOC concentrations and exposure information, this study will detail assessments conducted on both the carcinogenic and non-carcinogenic risks.

For the carcinogenic risk assessment of IVOCs, the Excess Cancer Risk (ECR) was generally used for the quantitative analysis. The ECR was characterized by the inhalation unit risk (IUR), which is the lifetime cancer risk per microgram, per cubic meter (µg m^−3^) of atmospheric pollutants. The ECR was calculated as per Equation (1):

(1) ECR=IUR×EC

When the ECR ≤ 10^−6^, the cancer risk is negligible; when 10^−6^ < ECR ≤ 10^−5^, there is a moderate cancer risk; when 10^−5^ < ECR ≤ 10^−4^, there is a high cancer risk; and when ECR > 1 × 10^−4^, there is a very high carcinogenic risk.

For the non-carcinogenic risk assessment of IVOCs, the Hazard Quotient (HQ, dimensionless) was used for the quantitative analysis, which is the ratio of the exposure concentration to the toxicity value. The HQ was calculated as per Equation (2):

(2) HQ=EC/(RfC×1000)

When HQ ≤ 1, there is a low non-carcinogenic risk; when 1 < HQ ≤ 2, there is a moderate non-carcinogenic risk; when 2 < HQ ≤ 3, there is a high non-carcinogenic risk; and when HQ > 3, there is a very high non-carcinogenic risk.

The exposure concentration (EC) was calculated as per Equation (3):

(3) EC=CA×ET×EF×ED/AT

The meanings of the parameters in the equations are shown in Table 1.

Health risk models inherently involve uncertainties. For example, exposure parameters may vary due to differences in the population characteristics, such as age, gender, and sensitivity. Exposure concentrations can also be uncertain due to factors like instrument precision, model accuracy, and variations in human activity patterns. Additionally, the toxicity values of pollutants often involve uncertainties because they are typically extrapolated from animal studies to humans. However, the technical specifications for health risk assessments of ambient air pollution do not provide error ranges [25], and it is difficult to calculate these uncertainties. As a result, it is difficult to clarify the margins of error in the model.

Among all the identified IVOC species, long-chain alkanes and sesquiterpenes are not classified as confirmed or potential carcinogens and have low carcinogenic risks. Thus, this study exempts the systematic evaluation of carcinogenic and non-carcinogenic risks for these two types of substances. According to the authoritative data from the IRIS, naphthalene (Nap) and benzo[a]pyrene (BaP) are the only substances with complete inhalation risk parameters, including IUR and RfC. Based on this, the actual measured average concentrations of Nap and BaP in each observation site provided in this paper were selected as the inputs in terms of the exposure parameters for the risk assessment.

## 3. Source Characteristics

### 3.1. Sources and Compositions

**Figure 2:**
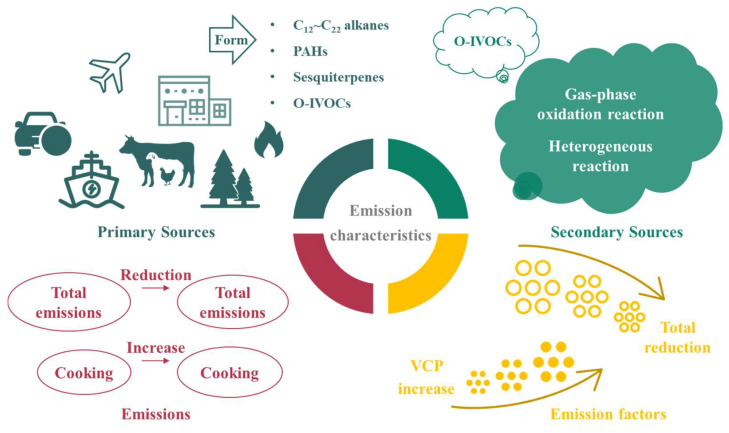
Conceptual diagram of the sources and emission characteristics of IVOCs.

IVOCs have extensive sources, mainly including primary emissions and secondary formations (Figure 2). Primary emissions are from anthropogenic and biogenic sources, involving fossil fuel combustion (e.g., motor vehicle exhausts, ship and aircraft emissions) [26,27,28], biomass combustion (e.g., wood and straw combustion, and garbage incineration) [29], industrial processes (e.g., chemical production and paint use) [30], catering [31], animal husbandry and plant emissions (e.g., terrestrial higher plants, aquatic plants, and phytoplankton) [32]. Secondary formations of IVOCs mainly occur via atmospheric chemical reactions. In gas-phase oxidation reactions, VOCs react with hydroxyl radicals (OH·), the ozone (O_3_), or nitrate radicals (NO_3_·) to form intermediate products like peroxy radicals (RO_2_·) and epoxides. These intermediate products further react to generate low-volatility compounds, which are partially converted into IVOCs. Additionally, RO_2_· can undergo auto-oxidation to form low-volatility peroxides and hydroperoxides, and these products partially belong to IVOCs [7]. In regard to heterogeneous reactions, IVOCs react with sulfate ions on the surface of acidic particulate matter to form low-volatility compounds, such as sulfate esters, or undergo photochemical reactions on particle surfaces or in droplets under light to generate low-volatility compounds [33,34], promoting the secondary formation of IVOCs.

The currently identified species of IVOCs cover long-chain alkanes (C_12_–C_22_), branched cyclohexanes, sesquiterpenes, PAHs, monocyclic aromatic hydrocarbons, phenolic compounds, ketones, esters, organic acids, and heterocyclic compounds. The primary components of IVOCs emitted during fossil fuel combustion are aromatic hydrocarbons and long-chain alkanes [35,36,37]. Compared to diesel vehicle exhausts, gasoline vehicle exhausts contain an obviously higher concentration of aromatic hydrocarbons and a relatively lower concentration of long-chain alkanes. It has been reported that important components of aircraft exhausts are low molecular weight PAHs and long-chain alkanes above C_12_. During biomass burning, a large amount of oxygenated IVOCs (O-IVOCs), PAHs, and long-chain alkanes are released, and the IVOCs emitted from different biomass sources vary [38,39]. For example, the pyrolysis of lignin in wood generates a large amount of methoxyphenols, and the combustion of deciduous plants commonly produces 2,6-dimethoxyphenol and guaiacol, while the burning of coniferous plants also emits guaiacol. The use of volatile chemical products (VCPs), such as paints, printing inks, adhesives, polishes, air fresheners, perfumes, and emulsions during industrial processes releases O-IVOCs, such as alcohols, esters, and ketones [40]. Biogenic emissions release sesquiterpenes, phenolic compounds, oxalic acid, long-chain alkanes, indole, and other compounds, with marine biogenic emissions releasing a higher amount of oxalic acid [41,42,43,44]. The secondary formation of oxalic acid may occur through the oxidation of long-chain dicarboxylic acids and glyoxylic acid emitted by marine biota via multiphase reactions. The photochemical oxidation of organic compounds contributes significantly to the formation of aldehydes, ketones, and organic nitrates, and, in the presence of ammonia (NH_3_), a large number of heterocyclic compounds are also generated [45]. In summary, long-chain alkanes and aromatic hydrocarbons typically originate from primary emissions associated with fossil fuels, as well as from biomass combustion processes. O-IVOCs (e.g., phenols, ketones, and esters) typically originate from biomass burning, industrial processes, and secondary oxidation. Organic acids and heterocyclic compounds are primarily derived from biogenic emissions and secondary formation.

### 3.2. Emission Characteristics

Currently, the research on IVOC emissions mainly focuses on fossil fuel combustion sources, biomass burning sources, cooking sources, and VCP usage sources. Pye and Seinfeld et al. used the primary organic aerosol (POA) emission inventory in the GEOS-Chem model, employing Nap as a surrogate for IVOCs, and estimated that the IVOC emissions from anthropogenic sources, biomass burning, and biofuel combustion in the year 2000 totaled 15 million tons of carbon per year [46].

In recent years, the overall emission of IVOCs in countries such as China and the United States has shown a downward trend, mainly attributed to the control of vehicle emissions [40,47]. Nevertheless, the emission of IVOCs from fossil fuel combustion remains significant. Vehicle exhausts are also an important source of IVOC emissions. The IVOCs emitted from gasoline and diesel vehicles account for a considerable proportion of VOCs, with diesel vehicles emitting much higher amounts of IVOCs than gasoline vehicles [35,48,49]. Trucks account for more than 70% of the total IVOC emissions. The IVOCs from global shipping emissions account for one-fourth of the IVOC emissions from land transportation and have already surpassed the total emissions from gasoline vehicles [50]. Wang et al. measured the IVOC concentration in Shanghai Yangshan Port before and after the G20 Summit in Hangzhou and found that the concentration of IVOCs decreased by 8% as a result of ship control measures, indicating that ships are also one of the most important sources of IVOC emissions [51]. From 2006 to 2020, the total IVOC emissions from motorcycles in China decreased from 197.19 Gg to 12.66 Gg [52]. However, IVOC emissions from certain specific sources, such as VCPs and cooking sources, have increased in recent years. Liu et al. established a full VOC emission inventory, revealing that industrial processes were the main contributors of IVOCs in the central China region in 2020, accounting for as much as 31.7% of the total IVOC emissions [53]. From 2015 to 2021, the emission of IVOCs from cooking sources in China increased, rising from a low level to 241,000 (135–374) tons per year. Commercial cooking is the main source of IVOC emissions, accounting for 66.2% of the total IVOC emissions, with Sichuan and Hunan cuisines contributing the most to the total cooking emissions [54].

Similarly, the emission factors (EFs) of IVOCs in countries such as China and the United States have generally decreased in recent years. There are many factors that affect the EFs of IVOCs from vehicles, including fuels, engines, driving conditions, vehicle age, accumulated mileage, and post-treatment technologies [55,56,57,58]. The fuel type and fuel quality directly affect the EFs of IVOCs, likely because a significant portion of IVOCs originate from incomplete fuel combustion [56]. For example, the EFs of IVOCs from large ships using heavy fuel oil are much higher than those from gasoline vehicles and are comparable to those from diesel vehicles [50]. Comparing the impacts of different engine types, it has been found that the EFs of IVOCs from two-stroke non-road mobile machinery are 4–5 times higher than those from four-stroke non-road mobile machinery. Qi et al. conducted field measurements of IVOCs emitted by non-road construction machinery in working, traveling, and idling conditions and found that the EFs of IVOCs ranged from 245.85 to 1802.19 mg kg^−1^ of fuel. Different operating modes significantly affect the EFs of IVOCs, with the EFs associated with the idling mode being 1.24 and 3.28 times higher than those associated with traveling and working modes, respectively [59]. Cycling conditions and post-treatment measures also influence the emission of IVOCs. In regard to low-speed cycling, the EFs of IVOCs are 6–23 times higher than those associated with high-speed cycling. In the same driving conditions, the EFs of IVOCs from diesel vehicles without post-treatment devices are 28 times higher than those from diesel vehicles equipped with diesel particulate filters (DPFs) [55]. The EFs of IVOCs also differ at different driving speeds. For example, only 7% of diesel fuel is consumed, but 30% of IVOCs are emitted during low-speed driving. The emission of IVOCs is much higher during hot starts than during cold starts [56]. Fuel quality can also affect the distribution of IVOC emissions. Based on the content of aromatic hydrocarbons, diesel fuel is divided into low, medium, and high aromatic diesel. Low aromatic diesel contains 9% aromatic hydrocarbons, medium aromatic diesel contains 12%, and high aromatic diesel contains 28%. The emission of PAHs via the exhaust of high aromatic diesel vehicles increases with the increase in the content of aromatic hydrocarbons, while the content of unresolved cyclic hydrocarbons decreases [11,55]. In addition, it has been found that the EFs of IVOCs from ships are related to the engine load, with the lowest emissions occurring at 75% load [60]. Although the EFs of motorcycles have decreased, their average EFs are still 7.78 times higher than those of China V-VI light-duty gasoline vehicles. It should be noted that the uncertainty in the IVOC emission data stems from the strong local characteristics and significant dynamic characteristics of the emission factors, the values of which change significantly depending on variable parameters, such as the fuel type and working conditions.

For biomass burning sources, the impact of different biofuels on IVOC emissions differ significantly. Qian et al. conducted field observations on IVOCs from 166 rural household solid fuels in eastern China and found that the average EFs of IVOCs from crop residues, firewood, and coal were 550.7 ± 397.9, 416.1 ± 249.5, and 361.9 ± 308.0 mg kg^−1^, respectively [61]. This indicates that the EFs of IVOCs from crop residue combustion are the highest, followed by firewood, while coal has relatively lower EFs. The combustion temperature and duration are also important factors affecting EFs. Higher combustion temperatures and longer durations promote more complete combustion, reducing the EFs of IVOCs; however, the high moisture content in biomass can reduce the combustion efficiency, leading to higher EFs for particulate matter and VOCs [39]. Due to the increased use of VCPs in recent years, the EFs of VCPs have significantly increased [47]. It has been reported that the EFs of VCPs are 1–2 orders of magnitude higher than those of gasoline vehicle exhausts [22].

Regional differences and variations in estimation methods contribute to uncertainties in emission estimates. For instance, the uncertainty range for IVOC emissions from biomass burning in the Pearl River Delta in China, calculated using the IVOCs/POA proportionality coefficient method, spans from −100% to 336%, while in the Yangtze River Delta in China, it ranges from −99% to 68%. For mobile sources in Guangdong Province in China, the overall uncertainty in IVOC emissions is between −54.7% and 144.9%. On a national scale in China, the estimated uncertainty for IVOC emissions falls within the range of −66% to 153% [31,62,63]. Despite existing reports on the emission characteristics of IVOCs, differences in emission characteristics and gas–particle partitioning have not yet been clearly distinguished and fully investigated. The simultaneous identification of gas-phase and particle-phase characteristics is a prerequisite for exploring the main sources and formation processes of IVOCs and is also of great significance for the development of IVOC control strategies. Moreover, understanding the trends in emissions and EFs overall and from specific sources of IVOCs is crucial to assess the air quality and climate impacts. Future research should focus on further identifying the EFs of IVOCs from different sources to improve the accuracy of emission inventories.

## 4. Ambient Levels

The total concentration of IVOCs varies in different environmental conditions. In Pasadena in the USA, the average concentration of IVOCs was measured as 6.3 ± 1.9 μg·m^−3^, comparable to that of OAs [11]. Notably, there was no significant difference in the IVOC concentrations between weekdays and weekends, indicating that other petroleum sources also contribute significantly to IVOC emissions. In Canada, indoor IVOC concentrations ranged from 14.4 to 69.0 μg·m^−3^, while outdoor concentrations ranged from 4.2 to 8.1 μg·m^−3^ [64]. The indoor IVOC concentrations were approximately six times higher than those outdoors, suggesting that indoor temperature and humidity conditions significantly affect the formation of IVOCs. Specifically, temperature was significantly positively correlated with the concentrations of branched alkanes and UCM, with higher temperatures leading to higher compound concentrations [65]. Relative humidity was significantly positively correlated with the concentrations of oxidized IVOCs (such as fatty acids), with high humidity potentially promoting the formation of these compounds [66].

In ambient air, several varieties of IVOCs can be detected. The atmospheric concentration of hexylcyclohexane is approximately 1.3 μg·m^−3^, while the emissions of C_12_–C_14_ n-alkyl cyclohexanes from diesel trucks range from 14.9 to 26.2 μg·km^−1^. These figures indicate that C_12_–C_14_ n-alkyl cyclohexanes have a certain concentration level in the atmosphere and account for a significant proportion of traffic emissions [18]. The average ambient concentration of IVOCs was (5.1 ± 0.8) μg·m^−3^ in Shanghai Yangshan Port in China [51], while it was (58.5 ± 27.0) μg·m^−3^ in winter and (6.8 ± 3.7) μg·m^−3^ in summer in urban Shanghai [10]. These data suggest that ship emissions contribute less to IVOCs than vehicle emissions and further confirm the positive correlation between temperature and IVOC concentrations. In low-temperature conditions, the volatility of many IVOCs decreases, causing them to exist more readily in the atmosphere in gaseous form.

**Figure 3:**
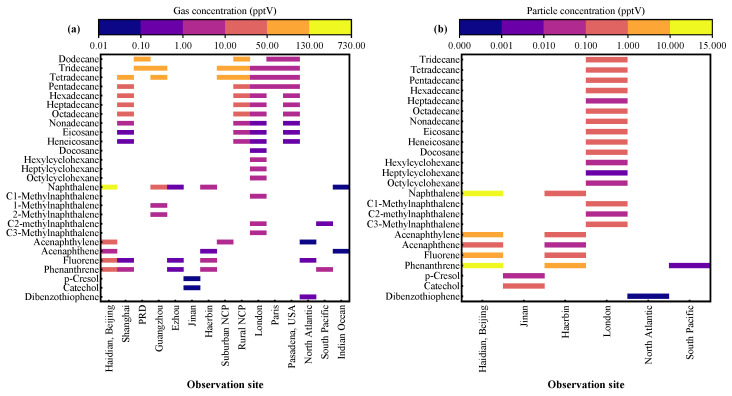
Atmospheric concentrations (pptV) of (**a**) gas-phase and (**b**) particulate-phase IVOCs at different observation sites [11,32,36,68,69,70,71,72,73,74]. PRD refers to the Pearl River Delta region in China, and NCP refers to the North China Plain (NCP) region in China.

Figure 3 summarizes the atmospheric concentrations of IVOC species, in both gas and particle phases, observed at different sites. The spatial distribution of gaseous PAHs shows that the Haidian District in Beijing, China [67], has significantly higher concentration levels than other urban areas. In regard to observations of gaseous long-chain alkanes in several typical regions in China (Shanghai [68], the Pearl River Delta urban agglomeration [69], Guangzhou [36], Ezhou [70], Jinan [71], Harbin [72], as well as rural and suburban areas of the North China Plain [69]), the concentration distribution consistently decreases with an increasing carbon number. In contrast, monitoring data from urban areas in Europe and America (London, UK [73], Paris, France [32], Pasadena, USA [11]) do not show such a carbon number-dependent concentration gradient. This may be attributed to the higher use of clean energy in European and American cities, significantly reducing the contribution of fossil fuel combustion sources to IVOCs. Notably, the concentration levels of gaseous PAHs in open ocean environments (the North Atlantic, South Pacific, and Indian Ocean) [74] are significantly lower than terrestrial observations, reflecting the low pollution characteristics of typical marine environments. Observation data indicate that, except for the higher values observed in the Haidian District, Beijing, the concentrations of particulate-phase IVOCs at other sites generally do not exceed 2 parts per trillion by volume (pptV), significantly lower than those of gaseous components (Figure 3). Particularly in open ocean areas (such as the North Atlantic and South Pacific), concentration levels are 1–2 orders of magnitude lower than those in terrestrial environments, reflecting the typical marine atmospheric background characteristics [75]. This also indirectly reflects that IVOCs primarily exist in the gas phase in the atmosphere, with their phase partitioning influenced by the characteristics of the emission sources.

In summary, the environmental concentrations of IVOCs exhibit significant spatiotemporal heterogeneity. It is mainly influenced by the combined effects of multiple factors, including source emission intensity, meteorological conditions, atmospheric chemical reactions, geographical spatial heterogeneity, and seasonal fluctuations [10,76,77]. Field observation data show that the spatiotemporal distribution characteristics of sampling sites (urban/rural, continental/oceanic, indoor/outdoor, heating season/non-heating season) are associated with IVOC concentration variations, resulting in distinct differences in observed values across different regions and time periods. The uncertainty in regard to IVOC measurement still remains, primarily due to the insufficient accuracy and sensitivity of existing equipment to identify the ambient composition of IVOCs, especially at low concentrations, and the presence of a large number of UCMs, which increases the difficulty in terms of the qualitative resolution and the quantitative measurement of IVOCs.

## 5. Contribution to SOA Formation

IVOCs make a significant contribution to the amount of SOA formation in the atmospheric environment, far exceeding that of traditional VOCs [78,79]. The experiment in the Pearl River Tunnel in October 2019 showed that SOAs generated from IVOCs emitted by vehicles were seven times that of VOCs [49]. Studies on vehicle emissions in California in the USA found that SOAs generated from IVOCs in gasoline and diesel vehicles accounted for 50% and over 95% of total SOAs, respectively [55,56]. From September to November 2018, high concentrations of long-chain alkanes were observed in the suburban Baoding area of the North China Plain and in urban Guangzhou in the Pearl River Delta. Their contributions to SOAs reached 9.4 ± 9.1% and 7 ± 8%, respectively, which were comparable to or higher than those of monocyclic aromatics and Nap to SOAs [69]. In July 2017, Huang et al. conducted field observations on a large cargo ship in Chinese waters and found that the estimated SOA formation from fuel emissions from the cargo ship was 546.5 ± 284.1 mg·kg^−1^ of fuel, with IVOC emissions from the ship contributing 98.9 ± 0.9% to the SOA formation [50]. In 2014, Zhao et al. conducted field observations in Pasadena in the USA, and found that although the concentration of IVOCs (6.3 ± 1.9 μg·m^−3^) accounted for only 7.4 ± 1.2% of the identified VOCs, their contribution to SOAs was as high as 57%, which was 4.75 times that of VOCs [11]. From 7 to 8 November 2014, Yang et al. conducted observations in Beijing, China, and found that the contribution of IVOCs to SOAs was as high as 82% of the total SOA concentration [80].

IVOCs not only significantly contribute to the formation of SOAs, but existing studies have also confirmed that SOA tracers can be used to effectively indicate IVOC species and their sources. This is of great significance for a deeper understanding of the behavior patterns of IVOCs in the atmosphere. However, corresponding research is still relatively limited, and available information is scarce. Current studies mainly focus on using SOAs as tracers to reveal information about IVOC sources. Specifically, levoglucosan, a major product of cellulose pyrolysis, has been widely recognized as a typical tracer for biomass burning; vanillic acid, derived from the pyrolysis of lignin in coniferous trees (e.g., pine), is a specific tracer for conifer combustion; syringic acid, originating from the pyrolysis of lignin in broadleaf trees (e.g., oak), accordingly serves as a tracer for broadleaf tree combustion [39]. In addition, four- and five-ring polycyclic aromatic hydrocarbons (PAHs) are considered tracers for light-duty gasoline vehicle exhausts, while three-ring PAHs are regarded as tracers for diesel vehicle emissions [81]. Although direct studies on SOAs indicating IVOC species are relatively lacking, it is now possible to clearly identify SOA tracers for naphthalene and other PAHs, as well as for sesquiterpenes, such as β-caryophyllene [82] (Table 2).

**Figure 4:**
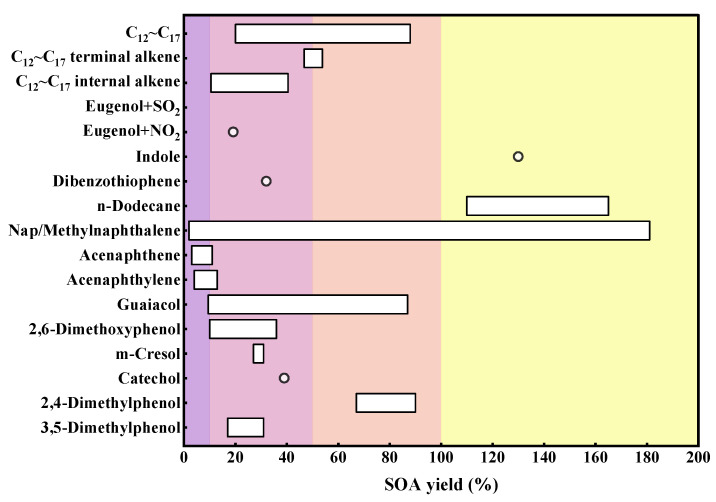
The SOA yield of certain components within IVOCs based on multi-laboratory studies. The white dots in the figure indicate individual values, while the columns illustrate a range of values. Purple indicates a yield of 0–10%, pink indicates a yield of 10–50%, orange indicates a yield of 50–100%, and yellow represents the yield exceeds 100%.

IVOCs exhibit significantly higher SOA yields compared to traditional VOCs, as shown in Figure 4. Laboratory studies have shown that the SOA yields of IVOCs are not only regulated by the baseline concentration, but are also closely related to multiple environmental variables. Key environmental regulatory factors include the oxidation pathways, the competitive mechanisms of nitrogen oxides (NOx), the surface catalytic effects of seed aerosols, temperature and humidity parameters, and the interfacial reaction mechanisms of coexisting inorganic gases. The SOA yields generated from the oxidation of C_12_–C_17_ long-chain alkanes by OH radicals range from 20 to 88% [83,84,85,86,87,88,89]. In contrast, the oxidation of n-dodecane initiated by chlorine atoms exhibits a super-stoichiometric characteristic, reaching a yield of 110–165% [90]. Dibenzothiophene, as a typical aromatic IVOC, maintains stable SOA formation efficiency at the level of 32% [91]. Systematic studies by the Loza team have shown that high NOx conditions can generally increase the SOA yields of different structural C_12_ alkanes [84]. This phenomenon is particularly prominent in the chlorine atom oxidation pathway of n-dodecyl to n-tetradecyl cyclohexanes, where the SOA yield reaches 90.14% in high NOx conditions, nearly double that of the 47.39% in low NOx conditions. This confirms the SOA formation advantage of this reaction pathway in urban atmospheric pollution scenarios [13]. Additionally, the introduction of dioctyl adipate seed can enhance the gas–particle partitioning efficiency, increasing the SOA yield by 5–15% [85,87]. Compared to low-humidity environments, elevated relative humidity (RH > 40%) can reduce the SOA yield by 11–55%, by increasing the aerosol liquid water content and altering the gas–particle partitioning coefficients [85,90]. The SOA derived from dodecane shows weak sensitivity to temperature, which is attributed to the extremely low volatility of its oxidation products, making it difficult for conventional temperature fluctuations to significantly change the condensation phase transition threshold of the products [86].

**Figure 5:**
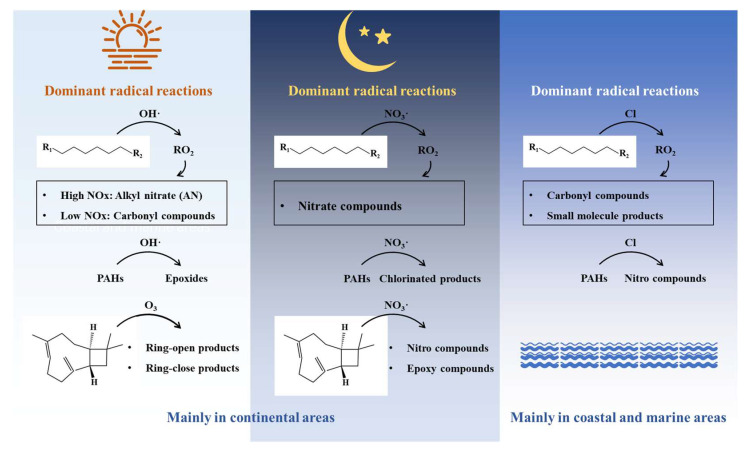
Oxidation mechanisms of common IVOCs species.

The process of IVOC oxidation leading to SOA formation involves complex chemical reaction mechanisms. Currently, research in this field remains relatively limited, with mechanism exploration primarily conducted through laboratory simulations. The current research on SOA formation pathways mainly focuses on the chain oxidation processes of long-chain alkanes, the cyclic structure transformation mechanisms of PAHs, and the formation pathways of oxidation products from sesquiterpenes (represented by β-caryophyllene) (Figure 5). Long-chain alkanes generate SOAs in the atmosphere through reactions with different oxidants, with the oxidation mechanisms varying depending on the type of oxidant [7]. The reactions of long-chain alkanes with OH· typically occur during the daytime, beginning with the abstraction of hydrogen atoms from alkane molecules by OH· to form alkyl RO_2_. In low NOx conditions, RO_2_ mainly reacts with HO_2_ to form peroxides (ROOH). ROOH can generate various products through reactions with OH· or photolysis, including carbonyl compounds and dihydroxy peroxides, which may further participate in the formation of atmospheric particulate matter. In high NOx conditions, RO_2_ mainly reacts with NO to form alkyl nitrates (AN) and alkoxy radicals (RO). RO can further decompose or react with O_2_ to form carbonyl compounds, which tend to remain in the gas phase, thereby reducing particle formation. The reaction of long-chain alkanes with Cl atoms mainly occurs during the day in coastal and marine regions. Cl atoms initiate the oxidation process by abstracting hydrogen atoms, generating RO_2_, which reacts with RO_2_ to form RO. RO mainly reacts with O_2_ to form carbonyl compounds or decomposes to produce small molecules with higher volatility, contributing relatively less to SOAs. Additionally, although the reaction of long-chain alkanes with NO_3_· is relatively slow, it is still significant due to the higher concentration of NO_3_· in the nighttime atmosphere. The products generated from the reaction of long-chain alkanes with NO_3_ radicals are mainly nitrate compounds, which have lower volatility and can significantly enhance SOA formation. PAHs primarily generate epoxides, nitro compounds, and chlorinated products through reactions with OH·, NO_3_·, and Cl atoms [92,93,94,95,96,97,98]. For example, acenaphthene reacts with OH· to form acenaphthenyl radicals, which can further react with O_2_ to generate peroxy radicals, ultimately forming low-volatility products that promote SOA formation. The reaction of acenaphthene with NO_3_· generates NO_3_-acenaphthene adducts, which can decompose to form 2,3-epoxyacenaphthene and 4,5-epoxyacenaphthene. The reaction of acenaphthene with Cl atoms produces chlorinated products, such as 2-chloroanthracen-1-one and 1-chloropyren-2-one. Sesquiterpenes like β-caryophyllene mainly generate cyclic and ring-opening products through reactions with O_3_ and NO_3_, such as β-caryophyllone aldehyde, β-norcaryophyllone aldehyde, β-caryophyllonic acid, and β-nocaryophyllonic acid [99]. These products further react in the atmosphere to form low-volatility substances, promoting SOA formation. Additionally, the products generated from the reaction of β-caryophyllene with NO_3_ include nitro compounds and epoxides, such as 1-nitroanthracene and 9-nitroanthracene. These products, with their high stability and low volatility in the atmosphere, significantly contribute to SOA formation.

The uncertainty in regard to model usage mainly stems from the insufficient accuracy of the emission factors, the inadequate temporal and spatial resolution of emission inventories, imperfect secondary oxidation reaction mechanisms, and inaccuracies in key kinetic parameters. Traditional air quality models have long underestimated SOA concentrations due to the neglect of IVOC emissions and oxidation mechanisms. By integrating IVOC emission inventories, optimizing kinetic parameters (such as the 2D-VBS), and incorporating aging mechanisms, the accuracy of SOA simulation results can be significantly improved. For example, Zhao et al. optimized smog chamber experimental parameters using a 2D-VBS box model and applied the scheme to a regional multiscale air quality model (CMAQ). They found that the introduction of aerosol aging and IVOC oxidation mechanisms increased OA and SOA concentrations in eastern China by approximately 40% and 10 times, respectively, greatly enhancing model performance [12]. Li et al., when simulating pollution in the Beijing–Tianjin–Hebei region during the winter of 2014, significantly improved the SOA simulation results after incorporating the IVOC emission inventory, with IVOCs contributing 40.1% to SOAs [100]. Wu et al. further corroborated this conclusion, with their model showing a 161% increase in SOA simulation concentrations in the Pearl River Delta region after introducing the IVOC emission inventory [31]. The optimization of kinetic parameters is a key aspect of model improvement. The 2D-VBS model, through its dual-dimensional (oxidation state and volatility) parameterization scheme, more accurately characterizes the gas–particle partitioning of IVOC oxidation products. In forested areas, the model achieved closure between simulated and measured SOA values after incorporating IVOC emissions and oxidation pathways [101], directly verifying the dominant role of IVOCs in natural-source SOA formation. Yang Wenyi et al. introduced long-chain alkane parameters obtained from smog chamber experiments into a regional model, finding that incorporating mechanisms for dodecane and other IVOCs increased the winter SOA simulation concentrations by 10–20 μg m^−3^, significantly reducing the deviation from field observations [80]. The introduction of aerosol aging mechanisms further enhanced the model’s ability to parse complex atmospheric processes. Zhao et al.’s simulations showed that the combined effect of aging mechanisms and IVOC oxidation pathways increased OA concentrations in eastern China from a 10-fold increase (considering only IVOCs) to 40%, revealing the nonlinear enhancement effect of multi-mechanism coupling on the simulation results [12].

## 6. Toxicity and Health Risk Assessments

**Figure 6:**
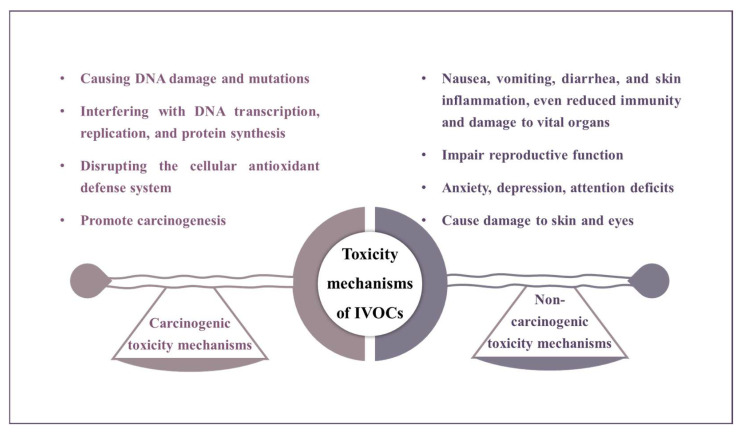
The toxicity mechanisms of IVOCs.

The toxicity mechanisms of IVOCs can be broadly categorized into carcinogenic and non-carcinogenic pathways (Figure 6). Certain IVOCs undergo metabolic activation in the body, generating reactive metabolites, such as epoxides, which are carcinogenic. These metabolites bind to DNA to form adducts, causing DNA damage, mutations, and, ultimately, cancer. Additionally, IVOC metabolites may interfere with DNA transcription, replication, and protein synthesis, disrupt gene expression related to cell growth and differentiation, and increase cancer risk. IVOCs can also induce oxidative stress, producing reactive oxygen species (ROS), damaging cellular antioxidant systems, and causing lipid peroxidation, protein oxidation, and DNA oxidative damage, further promoting carcinogenesis. In terms of non-carcinogenic risks, prolonged exposure to high concentrations of certain IVOCs can lead to acute toxicity, manifesting as nausea, vomiting, diarrhea, or skin inflammation, and, in severe cases, may impair immunity and damage vital organs. IVOCs can also induce DNA damage through mechanisms such as single- or double-strand breaks, base loss, guanine oxidation, thymine dimer formation, DNA adducts, and DNA crosslinking, potentially causing gene mutations and chromosomal abnormalities. Immune function may be suppressed by IVOCs, affecting immune cell activity and cytokine production, leading to immune suppression or imbalance. In regard to the reproductive system, IVOCs may disrupt hormone secretion (e.g., estradiol and testosterone), causing infertility or fetal malformations. The neurotoxic effects of IVOCs are associated with increased risk of anxiety, depression, and attention deficits, and may correlate with abnormal brain structure. Finally, certain IVOCs exhibit enhanced toxicity under UV or visible light exposure, converting into more toxic configurations, generating ROS, and causing cell membrane rupture, DNA strand breaks, and tissue damage in skin and eyes [102,103].

As representative IVOC species, Nap and BaP exhibit distinct toxicity profiles, as shown in Table 3. According to carcinogenicity classifications by the International Agency for Research on Cancer (IARC), BaP is definitively categorized as carcinogenic to humans (Group 1), whereas Nap is classified as possibly carcinogenic to humans (Group 2B), indicating stronger evidence for BaP’s carcinogenicity. In terms of long-term exposure risks, the reference concentration (RfC) for inhalation of BaP (2.0 × 10^−6^ mg m^−3^) is three orders of magnitude lower than that of Nap (3.0 × 10^−3^ mg m^−3^), and its inhalation unit risk (IUR, 6.0 × 10^−4^ (μg m^−3^)^−1^) is 17.6-fold higher than that of Nap (3.4 × 10^−5^), highlighting the substantially higher carcinogenic potential of BaP as a result of low-dose chronic inhalation exposure. Similarly, for oral toxicity parameters, BaP’s reference dose (RfD, 3.0 × 10^−4^ mg kg^−1^ day^−1^) represents only 1.5% of Nap’s value (2.0 × 10^−2^ mg kg^−1^ day^−1^), while its oral slope factor (CSFo, 1 mg kg^−1^ day^−1^) is 8.3 times greater than that of Nap (0.12), further confirming BaP’s significantly elevated carcinogenic risk via the oral route. In contrast, acute toxicity data reveal an opposing trend: the median lethal dose (LD50) for the intraperitoneal administration in mice demonstrates that Nap (150 mg kg^−1^) exhibits stronger acute toxicity than BaP (250 mg kg^−1^), suggesting that Nap may pose greater direct toxic effects in high-dose short-term exposure scenarios. Although both compounds share identical maximum contaminant levels (MCLs) in drinking water (0.2 μg L^−1^), combined analysis with oral slope factors indicates that BaP’s actual carcinogenic risk at equivalent concentrations far exceeds that of Nap. In conclusion, BaP dominates in terms of its chronic carcinogenic risks and should be prioritized in environmental and health regulations, whereas Nap’s toxicity management warrants greater focus on acute exposure contexts.

The human health risk assessment results for Nap and BaP across multiple regions (Table 4) show that the non-carcinogenic risk of Nap is generally within a safe range. With the HQ at all observation sites being below the safety threshold of 1, its non-carcinogenic risk is negligible. In terms of carcinogenic risk, significant spatial differences are observed across different geographical environments. Low carcinogenic risk levels (ECR < 10^−6^) are found in open areas, such as during winter in Harbin in China, the Pearl River Delta urban agglomeration, the North Atlantic, and the Indian Ocean. Medium carcinogenic risks (10^−6^ < ECR < 10^−5^) are observed in semi-enclosed areas, including rural north China, indoor environments in Germany, and the industrial area of Assiut, Egypt. Notably, the observation site in the high-density urban area of Haidian District, Beijing, China, has an ECR value exceeding 10^−5^, reaching a high-risk level. Data analysis reveals a triple-gradient characteristic in regard to Nap pollution risk. The risk in terrestrial systems is significantly higher than that in marine environments, with the average risk level in indoor microenvironments being more than double that of outdoor environments. Urban built-up areas also have risk values one order of magnitude higher than rural areas. This spatial differentiation pattern is likely closely related to the intensity of anthropogenic emissions and the characteristics of pollution sources, especially the regional distribution differences of traffic sources, industrial emissions, and residential sources. Similar conclusions have been drawn from PAH risk assessments in southern California, India, and several countries in South America [18,19,104]. It is recommended to focus on the coordinated control of indoor and outdoor urban environments and to establish a Nap risk early warning system based on source apportionment.

The carcinogenic risk value of BaP at the Svirsk site in Russia exceeds the high-risk threshold (10^−5^), while its non-carcinogenic risk is more than 10 times the safety threshold, indicating a complex health risk posed by BaP in this area. Similarly, significant risk accumulation is also observed in the Haidian District of Beijing, China, and the Assiut area in Egypt. In contrast, the ECR of BaP during winter in Harbin, Lianhu District of Xi’an, Jinan, Tokyo, Japan, and Gdynia, Poland, is all below 10^−6^, with a HQ below 1, meeting acceptable risk standards. Geographic analysis indicates that high-risk sites are mostly concentrated in urban industrial clusters (such as Svirsk in Russia and the Haidian District in Beijing, China), confirming the decisive role of industrial emissions and traffic sources in the spatial differentiation of BaP. It is recommended to establish a dynamic monitoring mechanism for BaP in key areas, focus on complex exposure scenarios, especially in urban built-up areas where ECR values exceed safety thresholds, and implement precise control strategies for BaP based on source apportionment and human health risk assessments.

## 7. Shortcomings and Prospects

The qualitative and quantitative analysis of IVOCs is gradually becoming a research hotspot in the field of environmental chemistry. Currently, numerous analyses of IVOCs have been conducted internationally. However, there are still some shortcomings in the research, mainly reflected in regard to the following three aspects. (1) Many IVOC components in environmental samples remain uncharacterized, especially due to the extensive presence of isomers and substituents, which significantly increases the detection difficulty. Existing analytical techniques (such as GC–MS, CIMS, and GC×GC–ToF–MS [77]) are still insufficient in terms of separation efficiency and detection sensitivity to meet the precise detection needs of IVOCs in complex environments. This results in limitations in regard to species identification rates and quantitative accuracy. (2) Although the macroscopic laws of IVOCs generating SOAs have been preliminarily revealed through field observations, smog chamber simulations, and model simulations, there is still a knowledge gap at the molecular level. For example, the atmospheric oxidation pathways of IVOC species, SOA tracer information, and the relevant physicochemical properties are not yet fully understood. (3) Current toxicological studies mainly focus on PAHs among IVOCs, while research on the exposure pathways, metabolic mechanisms, and combined toxic effects of emerging pollutants, such as oxygen-containing compounds (e.g., aldehydes and ketones) and heterocyclic compounds, is significantly lagging.

The qualitative and quantitative analysis of IVOCs is a complex and continuously evolving field, facing many challenges in the future. On the one hand, to achieve the successful quantitative analysis of ambient IVOCs and reduce uncertainties in IVOCs measurement, it is necessary to develop instruments with higher separation and resolution capabilities, especially online measurement instruments with a high temporal resolution. On the other hand, to further explore the qualitative characteristics of IVOC oxidation products and the health risks, and to reduce the uncertainties in model simulations, more comprehensive and in-depth research on their reaction mechanisms are needed, especially for aliphatic aldehydes, ketones, carboxylic acids, esters, ethers, and heterocyclic compounds in IVOCs.

## 8. Conclusions and Implications

### 8.1. Conclusions

This study systematically reviews the latest research progress on IVOCs, both domestically and internationally, summarizes the research advancements in terms of source emission characteristics, ambient concentration levels, and contributions to SOA formation of IVOCs, and conducts a health risk assessment of typical IVOC species, revealing their key roles in regard to atmospheric chemistry and public health.

The sources of IVOCs are diverse, and their chemical composition shows significant source-dependent characteristics. In recent years, although the overall emission of IVOCs has shown a downward trend, the emissions and emission factors associated with certain sources (e.g., VCPs) have increased. The concentration of IVOCs exhibits significant spatiotemporal heterogeneity and phase distribution characteristics. Urban areas generally show higher IVOC concentrations, with indoor concentrations being significantly higher than outdoor levels. In China, the concentration of long-chain alkanes shows a carbon number-dependent decreasing trend, which is not observed in European and American cities. Marine concentrations of IVOCs are relatively lower compared to terrestrial environments.

As important precursors of SOAs, IVOCs make a significant contribution to their formation and yield that far exceeds that of traditional VOCs, especially in traffic-dense areas and high-emission urban zones. SOA tracers for species such as β-caryophyllene and naphthalene have been identified. The SOA yields of IVOCs are generally high and are regulated by environmental variables, such as oxidation pathways, NOx concentrations, seeds, temperature and humidity, and coexisting inorganic gases. The main pathways for IVOCs to generate SOAs are through oxidation by OH·, NO_3_·, and Cl atoms. The oxidation pathways of long-chain alkanes are mainly regulated by NOx concentrations; PAHs generate epoxide, nitro, and chlorinated products through radical addition; and sesquiterpenes react with O_3_ or NO_3_ to form epoxides and nitro compounds. Owing to the above key role in SOA formation, integrating IVOC emission inventories has significantly improved the SOA simulation accuracy of regional air quality models.

Health risk assessments indicate that the carcinogenic risk of Nap should be prioritized for control purposes, while its non-carcinogenic risk management priority can be moderately reduced. In contrast, BaP requires combined risk assessment and tiered management of both its carcinogenic and non-carcinogenic effects. The carcinogenic and non-carcinogenic risks of Nap and BaP show significant spatial differentiation, with high-risk hotspots concentrated in dense urban areas and industrial clusters.

This review integrates the research findings along the entire chain of “emission–exposure–transformation–risk” to systematically analyze the pollution characteristics and environmental impact of IVOCs, filling some gaps in the understanding of IVOCs. Future research is expected to focus on the development of high-resolution online detection technologies, in-depth analysis of multiphase reaction mechanisms, and comprehensive assessment of human health risks.

### 8.2. Implications

Several governments have issued a series of policies to promote the comprehensive management of volatile organic compounds, such as the “14th Five-Year Plan” for Integrated Energy Conservation and Emission Reduction and the National Volatile Organic Compound Emission Standards for Consumer and Commercial Products (40 CFR 59). These policies unintentionally include IVOC compounds, such as VCPs. However, there are no regulatory policies specifically targeting IVOCs. Identifying the emission sources of IVOCs is fundamental to devising reduction policies. For IVOC emission sources such as vehicle exhausts, industrial activities, and biomass burning, corresponding reduction measures can be established, like enhancing vehicle emission standards and intensifying industrial emission monitoring. Monitoring and analyzing ambient IVOC concentrations across different regions helps to identify key areas for governance, like large cities or industrial zones with high IVOC levels that require stricter control mechanisms. Assessing the potential health impact of IVOCs helps determine which species pose the greatest health risks, allowing for prioritized emission control of these compounds. The findings from this review can inform the development of targeted policies for IVOC prevention and control.

## Figures and Tables

**Table 1:** Meanings of relevant parameters used in human health risk assessment methods.

Parameters	Definitions	Values	Units
CA	Contaminant concentration in air		μg·m^−3^
ET	Exposure time	3.7	h/d
EF	Exposure frequency	365	d/y
ED	Exposure duration	78.6	y
AT	Averaging time	78.6 × 365 × 24	h
RfC	Reference concentration		mg·m^−3^

**Table 2:** Currently identified SOA tracers capable of indicating species of IVOCs.

IVOC Precursors	SOA Tracers	Indicator for Sources
Nap	4-nitrophthalic acid	PAHs
	4-nitro-1-naphthol	
	2,4-dinitro-1-naphthol	
	Phthalic acid	
β-caryophyllene	β-caryophyllinic acid	Sesquiterpenes

**Table 3:** A toxicity comparison of Nap and BaP.

Toxicity Parameters	Nap	BaP	Units
IARC Carcinogenic Classes	Possibly carcinogenic to humans	Carcinogenic to humans	
Reference Concentration (RfC)	3.0 × 10^−3^	2.0 × 10^−6^	mg m^−3^
Inhalation Unit Risk (IUR)	3.4 × 10^−5^	6.0 × 10^−4^	(μg·m^−3^)^−1^
Reference Dose (RfD)	2.0 × 10^−2^	3.0 × 10^−4^	mg kg^−1^ day^−1^
Oral Slope Factor (CSFo)	0.12	1	mg kg^−1^ day^−1^
Maximum Contaminant Levels (MCLs)	0.2	0.2	μg L^−1^
Median Lethal Dose (LD50) (Intraperitoneal, Mouse)	150	250	mg kg^−1^

**Table 4:** Related values of Nap and BaP in IVOC species for human health risk assessment.

Nap (ng·m^−3^)	Sampling Location	ECR (×10^−6^)	HQ	BaP (ng·m^−3^)	Sampling Location	ECR (×10^−6^)	HQ
3879.4 [67]	Haidian, Beijing	20.33	0.20	52.58 [67]	Haidian, Beijing	4.86	4.05
38.5 [72]	Harbin Winter	0.20	0.00	4.61 [72]	Harbin Winter	0.43	0.36
174 [69]	PRD	0.91	0.01	1.33 [105]	Lianhu, Xi’an	0.12	0.10
224 [69]	Rural NCP	1.17	0.01	2.04 [106]	Jinan, China	0.19	0.16
596 [107]	Classroom, German	3.12	0.03	155 [108]	Svirsk, Russia	14.34	11.95
378.6 [109]	Assiut, Egypt	1.98	0.02	73.6 [109]	Assiut, Egypt	6.81	5.67
41 [74]	North Atlantic	0.21	0.00	2.91 [110]	Tokyo, Japan	0.27	0.22

PRD refers to the Pearl River Delta region in China, and NCP refers to the North China Plain (NCP) region in China. The IUR of Nap is 3.4 × 10^−5^ (μg·m^−3^)^−1^ and the RfC is 3.0 × 10^−3^ mg·m^−3^; the IUR of BaP is 6.0 × 10^−4^ (μg·m^−3^)^−1^ and the RfC is 2.0 × 10^−6^ mg·m^−3^.

## Data Availability

No new data were created or analyzed in this study. Data sharing is not applicable to this article.
